# Chromitite layers indicate the existence of large, long-lived, and entirely molten magma chambers

**DOI:** 10.1038/s41598-022-08110-6

**Published:** 2022-03-08

**Authors:** Rais Latypov, Sofya Chistyakova, Stephen J. Barnes, Belinda Godel, Gary W. Delaney, Paul W. Cleary, Viktor J. Radermacher, Ian Campbell, Kudakwashe Jakata

**Affiliations:** 1 School of Geosciences, University of the Witwatersrand, Johannesburg, South Africa; 2 CSIRO Mineral Resources, Kensington, Perth, WA 6151 Australia; 3 CSIRO Data61, Clayton South, VIC 3169 Australia; 4 Evolutionary Studies Institute, University of the Witwatersrand, Private Bag 3, Perth, 2050 South Africa; 5 Australian National University, Canberra, ACT 0200 Australia; 6 European Synchrotron Radiation Facility (ESRF), Grenoble, France

**Keywords:** Solid Earth sciences, Geochemistry, Geology, Mineralogy, Petrology, Volcanology

## Abstract

The classical paradigm of the ‘big magma tank’ chambers in which the melt differentiates, is replenished, and occasionally feeds the overlying volcanos has recently been challenged on various grounds. An alternative school of thought is that such large, long-lived and largely molten magma chambers are transient to non-existent in Earth’s history. Our study of stratiform chromitites in the Bushveld Complex—the largest magmatic body in the Earth’s continental crust—tells, however, a different story. Several chromitites in this complex occur as layers up to 2 m in thickness and more than 400 kms in lateral extent, implying that chromitite-forming events were chamber-wide phenomena. Field relations and microtextural data, specifically the relationship of 3D coordination number, porosity and grain size, indicate that the chromitites grew as a 3D framework of touching chromite grains directly at the chamber floor from a basaltic melt saturated in chromite only. Mass-balance estimates imply that a few km thick column of this melt is required to form each of these chromitite layers. Therefore, an enormous volume of melt appears to have been involved in the generation of all the Bushveld chromitite layers, with half of this melt being expelled from the magma chamber. We suggest that the existence of thick and laterally extensive chromitite layers in the Bushveld and other layered intrusions supports the classical paradigm of big, albeit rare, ‘magma tank’ chambers.

## Introduction

For over a century, the classical paradigm of magma chambers has underpinned all models of the Earth’s magmatism. This paradigm envisages a magma chamber as a large body of the molten, long-lived, and slowly fractionating magma (‘a big magma tank’) enclosed in crustal rocks^1–9^. In recent years, this classic view of a magma chamber has been challenged by the view that largely molten ‘big tank’ magma chambers are either very short-lived or never existed in Earth’s history^10–20^. Most volcanologists have abandoned the classic paradigm because geophysical surveys have failed to detect any present-day eruptible magma bodies in the Earth’s crust^12,15^. As an alternative, they proposed the existence of transcrustal mushy systems (including mushy reservoirs for mafic layered intrusions^12^) that are formed in the crust from numerous coalescing intrusions. These transcrustal systems contain only small melt lenses that are produced by compaction^11,14^ or tectonic destabilization^12,15^ of the crystal mush and exist for only a very short period of time before accumulating and erupting as lavas on the Earth’s surface^11–15^. Some petrologists have also proposed on the evidence of out-of-sequence zircon geochronological data^16,17,20^ that mafic plutons do not require the existence of large magma chambers^19,21,22^ but are rather produced as a stack of randomly-emplaced sills, with successive crystal-rich pulses often invading pre-existing cumulates^16–20,22,23^. In this study, we present field and microtextural data on chromitites from the Bushveld Complex whose formation require many times their own volume of magma to supply the key component, chromium (Cr). We argue on textural and mass balance evidence that these chromitite layers formed by in situ crystallisation and that their origin can be best understood in the frame of the classical concept of the ‘big magma tank’ chambers^1–9,24^.

### The enormous extent of chromitite layers

**Figure 1:**
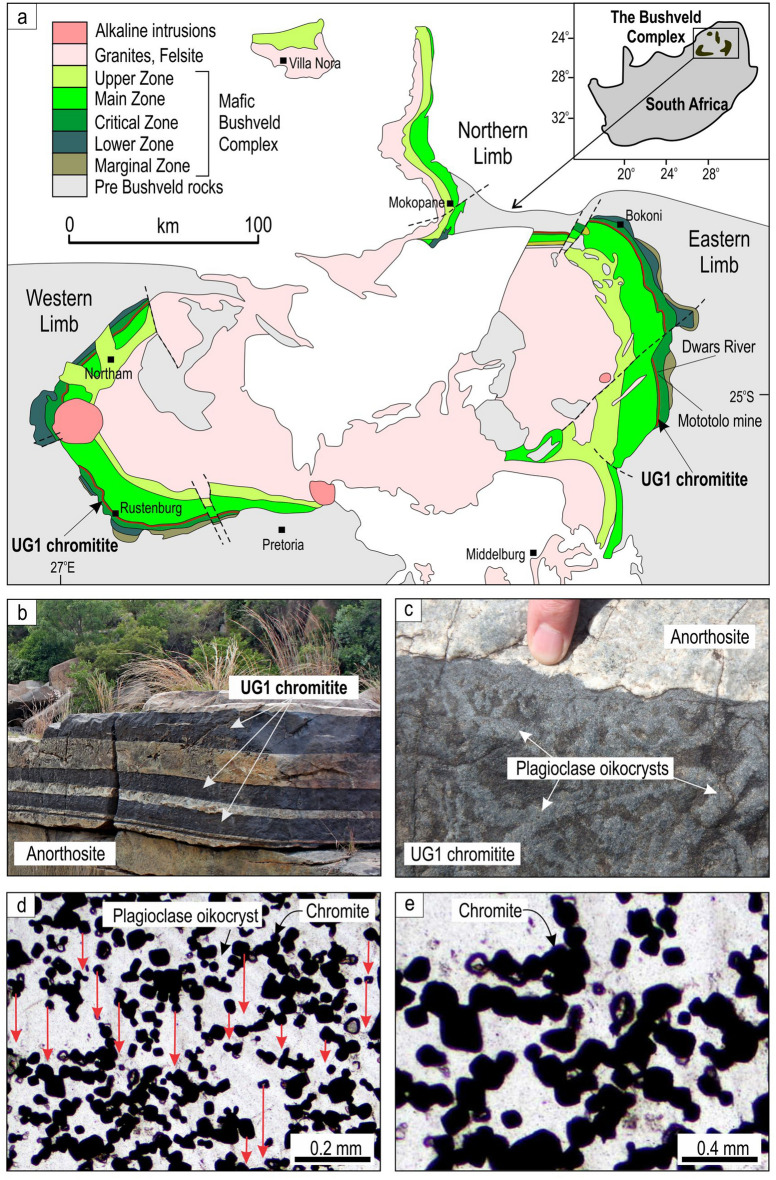
A notable lack of textural evidence for gravity settling of chromite crystals within a UG1 chromitite of the Bushveld Igneous Complex, South Africa. (**a**) Location and generalized geological map of the Bushveld Complex emphasizing its enormous size. Note that this is just an erosional remnant of the complex so that its original size was even larger. The immense lateral extent of the chromitite layers (> 350–400 km) in this complex is illustrated here by the UG1 chromitite that occurs at the top of the Critical Zone. Also indicated are places from which the studied samples were obtained. The map is compiled from many open sources^5,16,25,28,30,31,34^ and is prepared using CorelDRAW (version 18.1.0.690). (**b**) Panoramic view of a few sublayers of the UG1 chromitite in the anorthosite footwall at the Dwars River, Eastern Limb. **c,** A close-up photograph of the top part of the UG1 chromite sublayer. Note large plagioclase oikocrysts enclosing numerous small crystals of cumulus chromite (chadacrysts). (**d,e**) Photographs of a thin-section (under plane polarized light) of the UG1 chromitite showing isolated chromite grains and their loose clusters enclosed by a single large oikocryst of plagioclase. Red arrows emphasize that chromite grains show no tendency to gravitate downwards despite a high porosity of the framework (~ 65 vol.%). Also note that most chromite crystals in (**e**) are touching each other along crystal faces. Sample HX-07–153.33, Mototolo mine, Eastern Limb.

The 2.05 Ga Bushveld Complex in South Africa (Fig. 1a) is the largest mafic–ultramafic layered intrusion in Earth’s crust; totalling to about 600,000 km^3^ of igneous rocks^25,26^. The complex consists of several parts, the western, eastern and northern limbs being the largest, and is subdivided stratigraphically into five major units—the Marginal, Lower, Critical, Main, and Upper Zones, comprising a total thickness of 7 to 9 km^25,26^. The Bushveld Complex contains > 80% of the Earth’s known chromium resources^27^, an element critical to improving the material properties of steel, making this magmatic body an object of perennial study. The chromium is hosted within 14 principal layers of massive chromitites, mostly confined to the Critical Zone^28,29^. Three major groups of chromitites are recognised: the Lower (LG1 to LG7); Middle (MG0 to MG4); and Upper Groups (UG1 to UG3)^29^. The thickness of individual chromitite layers ranges from a few decimeters to 2 m. Mining activities have allowed most of these layers to be traced across the entire Bushveld Complex^29^. Remarkably, the vertical distribution of platinum-group elements across some of these chromitites are nearly identical in places that are separated laterally by over 300 km^28^. The vast lateral extent and mineralogical uniformity of chromitite layers indicate that the process responsible for their formation has been working synchronously over lateral distances of up to 400 km (e.g., UG1 in Fig. 1a) to produce continuous, uniform blankets of chromitite.

### Field and textural evidence for in situ growth of chromite

The nature of intrusion-wide chromite-forming processes can be constrained from field and textural features of massive chromitite layers such as the 2 m-thick UG1 chromitite—the thickest and the best exposed layer in the entire complex (Fig. 1b). This chromitite shows remarkable field relationships with its respective footwall rocks. In addition to its occurrence on the planar portions of the chamber floor, this chromitite develops within potholes, roughly circular depressions in which footwall rocks are missing due to magmatic erosion^30,31^. In these areas, the planar UG1 chromitite that occurs along the periphery of the potholes commonly passes, often without any apparent changes in thickness and texture, into the steeply dipping, subvertical and even overhanging UG1 chromitite in the interior of potholes^30,31^ (Extended Data Fig. 1). This field observation is not consistent with the formation of the UG1 chromitite, both on the planar and overhanging portions of the chamber floor, by processes involving gravity-induced settling of chromite through either the resident melt^32–35^ or a crystal-rich mush^36,37^. The simplest alternative mechanism is in situ crystallization of chromite from a chromite-only-saturated melt^30,38^. This process implies that all crystals nucleate and grow in situ, i.e., directly along the roof, walls and floor of the magma chamber. In our case, the nucleation is supposed to happen heterogeneously on the pre-existing crystals growing on the chamber floor^8,39,40^. This process allows a continuous blanket of chromitite to cover all the planar and irregular margins, even the places where gravity-settling of chromite grains seems to be physically impossible (i.e., “gravity-settling shadows” in which dips are overturned^30,31^) (Extended Data Fig. 1).

An intriguing challenge here is to decipher how in situ growth of chromite is recorded in the texture of massive chromitites themselves. We have re-visited the UG1 chromitite from the classical Dwars River locality^41^ (Fig. 1b) where it is composed of 25–50 vol% of cumulus chromite that occurs as separate idiomorphic grains or clumps of grains that are smaller than 0.1 mm in size (Fig. 1c,d). The small grain sizes are emphasized by the fact that 1 cm^3^ of the rock contains, at least, 500,000 individual chromite crystals. The chromite grains are enclosed within much larger oikocrysts of plagioclase (up to 5–10 cm in size) that are clearly visible in outcrops (Fig. 1c). The traditional interpretation of such layers in the frame of gravity settling models is that chromite was the first to settle on the chamber floor^32–35^ followed, after some period of post-depositional cooling, by in situ growth of plagioclase oikocrysts from the interstitial melt in a mushy chromitite. An important point is that settling chromite grains have enough time to reach the chamber floor and continue growing there. The subsequently forming oikocrysts may capture and armour chromite from experiencing further growth, producing snapshots of an immature solidification front.

A close look at the UG1 texture (Fig. 1d) raises, however, a simple but fundamental quandary. Chromite is almost twice as dense as a basaltic melt (4,800 kg/m^3^ and 2,600 kg/m^3^, respectively) and is expected to settle to the chamber floor in a random closely-packed lattice in which all adjacent chromite grains are touching each other. However, this is not the case as chromite occurs as individual grains and clumps of grains that seemingly ‘suspended’ within plagioclase oikocrysts (Fig. 1d). This observation leads to a critical question: why have the chromite grains/clumps failed to sink towards the chamber floor despite being much denser than the host melt? A potential clue to this puzzle is that the chromite grains in the UG1 layer appear to be arranged in 3D chain-like aggregates^42,43^.

### Three-dimensional framework of chromite crystals

**Figure 2:**
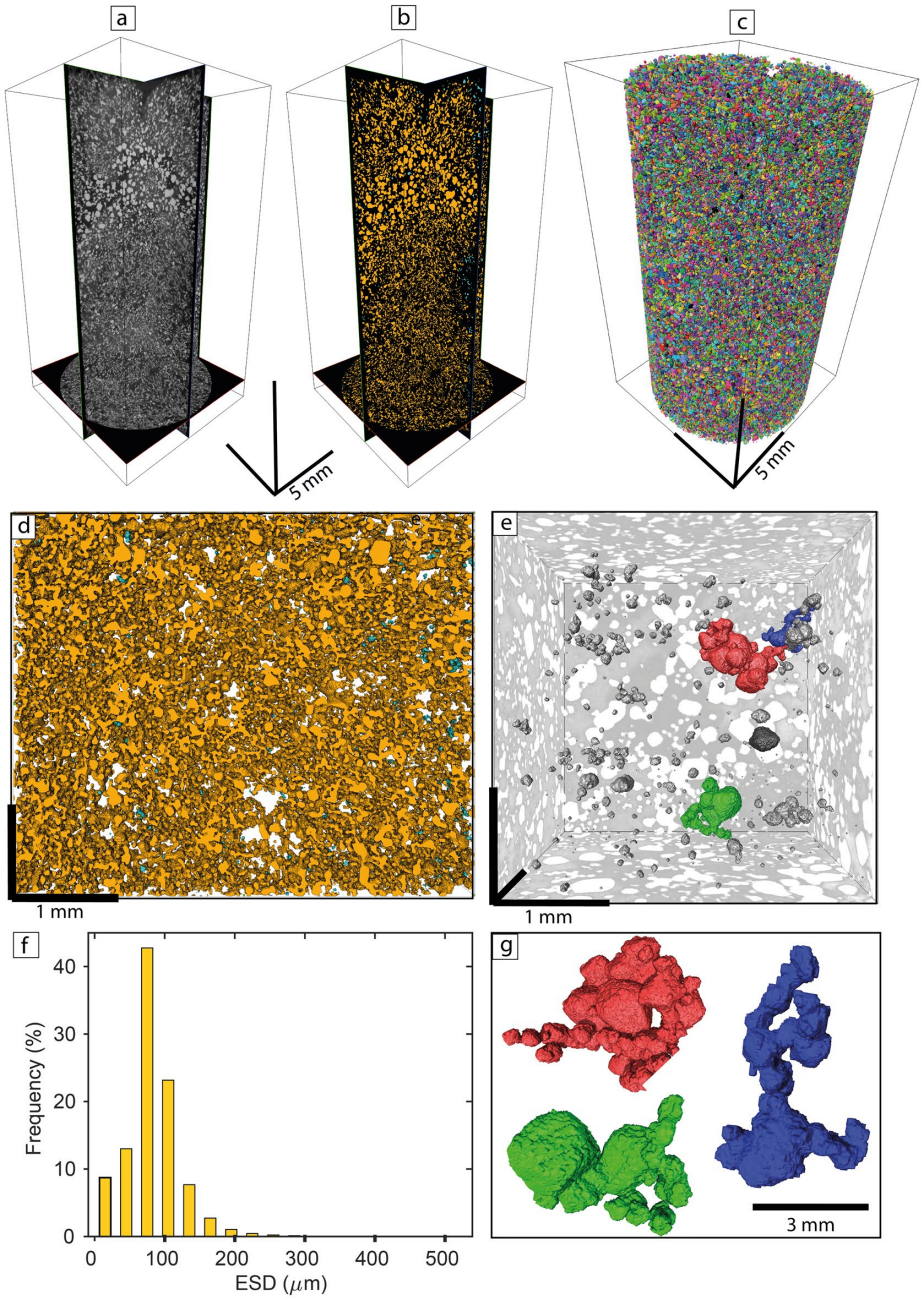
Results of high-resolution X-ray computed tomography revealing that nearly all chromite grains in UG1 chromitite are interconnected within a continuous 3D framework. (**a)** Three orthogonal slices virtually cut through the UG1 sample (HX-07–153.33, Mototolo mine, Eastern Limb) showing chromite in light grey;(**b**) Segmented chromitite showing how a single interconnected network (coloured in yellow) covers the entire sample volume; (**c**) Volume rendering of chromite grains displayed using a false 256 colour scale; (**d**) Expanded view of a volume of interest showing details of the interconnected chromite network (orange) and the isolated chromite grains (in cyan); (**e**) and (**g**) Details of selected chromite cluster morphologies within the large interconnected chromite network where only a small number of grains are coloured to improve visibility. Note that most chromite crystals are touching each other along crystal faces; (**f**) Histogram showing the size distribution of chromite grains in the sample (ESD: equivalent sphere diameter).

The analysis and quantification of chromitite in three-dimensions (3D) using high-resolution X-ray computed tomography (HRXCT) revealed that nearly all chromite grains (97 vol%) from the UG1 chromitite are interconnected to form a single continuous 3D framework composed of many thousands of grains that extend across multiple plagioclase and pyroxene oikocrysts (Fig. 2; Extended Data Fig. 2; Supplementary Video 1). We can now consider whether these UG1 microstructures (Fig. 2) could be generated by random loose packing of non-interacting particles, i.e., from small independent chromite grains settling from a basaltic melt by gravity settling or kinetic sieving^44^.

Theoretical microstructures of random mechanical crystal packs can be predicted by packing theory and characterised by two properties: the packing density (inverse of porosity); and the statistical distribution of coordination numbers, i.e., the number of grains of the same mineral that each grain touches. Quantification of clustering and chain formation has typically used assumptions of constant grain sizes^45^ but these parameters are known to be sensitive to the particle size distribution^46^. No observations or simulations have been made to date using the characteristic negative log-linear particle size distributions of crystals found in cumulates. To address this gap, we measured chromite particle sizes and coordination number distributions from the segmented HRXCT scan of the UG1 chromitite (Methods, Supplementary Data 1), choosing a volume within which chromite grains are primarily enclosed within plagioclase or pyroxene oikocrysts. This is to eliminate possible effects on microstructure caused by later recrystallisation and annealing. We then compared the results with those of a Discrete Element Method^59,60^ computer simulation of a random loose packing, generated by simple settling, of an assemblage of crystals with the same size distribution as the UG1 sample (see Methods for details of the modelling technique).

**Figure 3:**
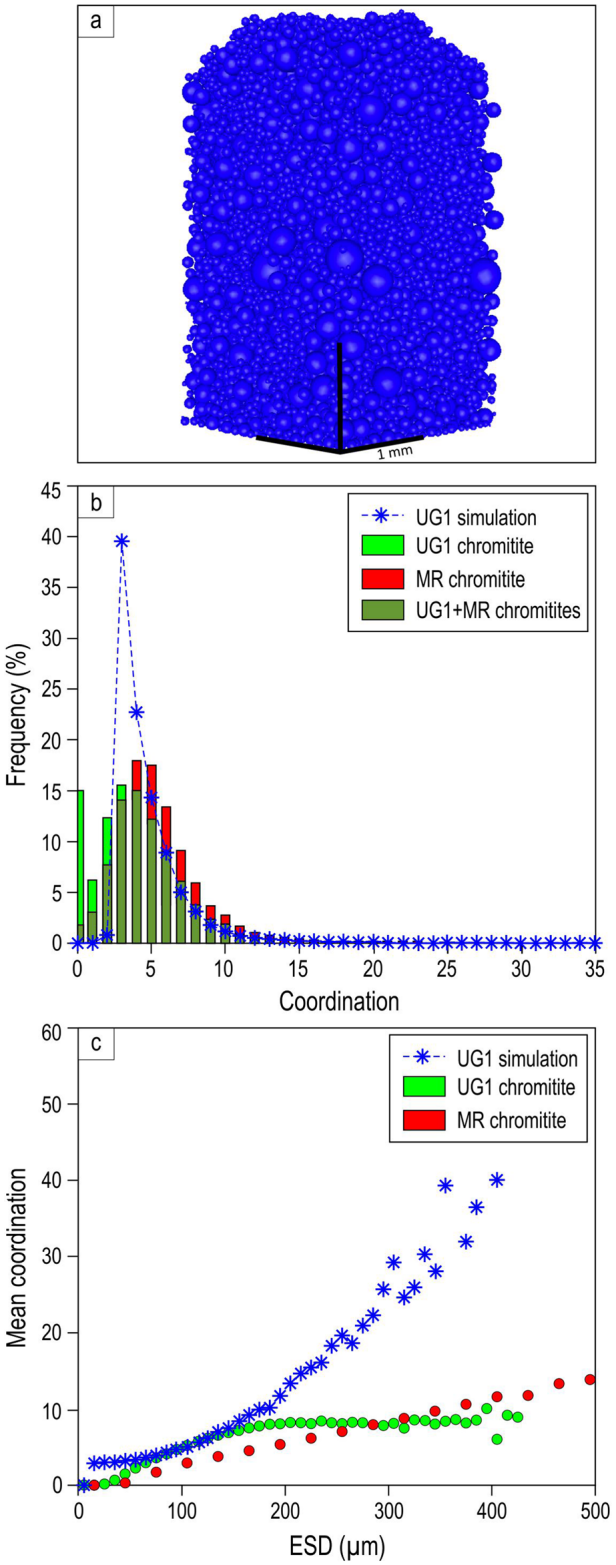
Results of numerical simulation compared to the observed natural data for the UG1 chromitite showing the contrasting relationship between grain size and coordination number between the randomised loose packing simulation (using the observed UG1 grain size distribution) and the two natural samples, the UG1 chromitite and MR overhanging chromitite. (**a**) 3D perspective view of a simulation pack of spheres having the same size distribution as chromite grains in the UG1 chromitite; (**b**) Histogram showing the distribution of coordination number in UG1 simulation, UG1 chromitite and MR overhanging chromitite; (**c**) Plot showing mean coordination number (i.e., the number of other chromite grains each grain touches) of all grains within each size range bin, as a function of size range of chromite grains in the UG1 simulation, UG1 chromitite and MR overhanging chromitite (ESD: equivalent sphere diameter). The separation of touching chromite crystals in 3D for (**b**) and (**c**) was done using Avizo2020.1 and Matlab software (R2019b).

Results show two distinct differences between the synthetic pack and the natural sample (Fig. 3): firstly, the packing densities are greatly different, being much higher in the synthetic pack than in the natural sample (60% vs. 27%). Secondly, the distribution of coordination numbers is significantly different (Fig. 3b,c). In the random pack, coordination number increases exponentially with the grain size. This happens because, for geometrical reasons, larger grains have a larger surface area and hence are likely to be in contact with a larger number of smaller grains filling space between them. In the natural UG1 data set, however, the coordination number flattens out and remains roughly constant at about 10 for grains larger than 150 microns in size (noting that only a small proportion of the total number of grains falls in this size range). This results from the chromite grains forming an open cage-like or chain-like structure where gaps in the framework are not occupied by other grains, causing lower coordination numbers at larger grain sizes. Furthermore, the natural sample contains a significant proportion of isolated or nearly isolated grains with coordination numbers of 0, 1 or 2; these are absent in the simulation. We conclude that (a) the low packing density, (b) the presence of isolated individual grains not supported by contact with any other chromite grains, and (c) the relationship between coordination number and crystal size in the UG1 chromitite are not consistent with random mechanical accumulation of non-interacting chromite grains, be it crystal settling in a melt^32–35^ or kinetic sieving in a crystal mush^16,36,37^.

There remains a possibility that chromite may settle in the form of chromite chains/clusters produced either by heterogeneous nucleation against chromite grains suspended in the convecting melt^39^ or physical collision of isolated chromite crystals ‘swimming together’ in this melt (i.e. “synneusis”)^47^. The accumulation of such clustered chains on the chamber floor would give rise to the formation of a continuous 3D framework of touching chromite crystals (Figs. 1–3). However, this scenario finds no support in the field observations: neither individual grains nor clustered chains can settle onto overhanging margins of potholes^30,31^ (Extended Data Fig. 1). Sidewall crystallisation thus indicates that the 3D chromite framework has most likely crystallized in situ, i.e., directly at the chamber floor. This may happen by heterogeneous/self-nucleation^8,39,40^ of chromite grains on the floor cumulates^30,31^. To develop this point further, we also compare the microstructure of a Merensky Reef chromitite seam that occurs on vertical to overhanging sidewalls of potholes (Extended Data Figs. 3 and 4; Supplementary Video 1), taken as a definitive example of a microstructure that could only have developed *in situ*^31,48^ (Fig. 3c).

**Figure 4:**
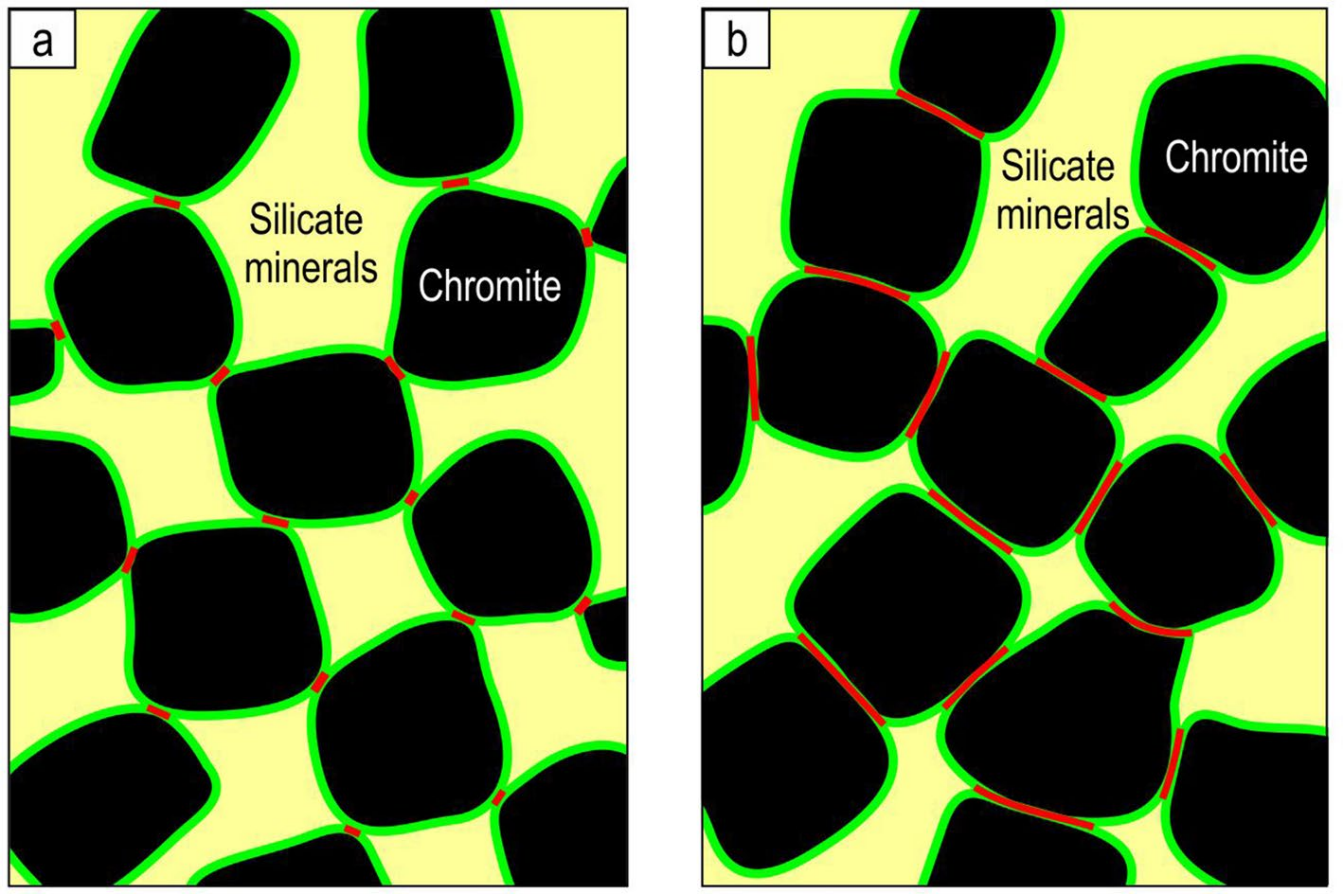
Two hypothetical cases of 2D sections through 3D images showing subdivision of chromite boundaries into chromite-to-silicate minerals (green) and chromite-to-chromite (red). (**a**) Case for chromite grains primarily in point-to-point contact (low ratio of red to green), (**b**) Case for chromite grains mainly in face-to-face contact (high ratio). Observed ratio in the studied sample of the UG1 chromitite (HX-07–153.33, Mototolo mine, Eastern Limb) is 0.34, indicating that chromite grains are mostly touching each other along crystal faces. The figure is prepared using CorelDRAW (version 18.1.0.690).

The packing density is likewise lower than the random loose packing simulation (53% vs 60%) (Supplementary Data 1). The coordination number vs grain size curve for this seam shows a steady linear increase with grain size, but, like the UG1 chromitite, has systematically lower coordination number values than the simulation along the entire length of the trend (Fig. 3c). Significantly, the packing and coordination number characteristics of the UG1 chromitite are much closer to those of the in situ crystallised sidewall Merensky Reef chromitite than to the random packing simulation. We deduce that the relatively low coordination number values and low packing densities of both natural samples are the result of in situ growth of chromite chains or cages by heterogeneous self-nucleation. We also conclude that chromite grains (Fig. 1d) are not able to settle freely towards the chamber floor simply because they are all bound together in self-supporting frameworks attached to the floor.

The point was raised in review as to whether chains/framework of chromite grains could remain intact during accumulation. The structural integrity of crystal chains depends on the nature of the contacts between the grains. This was investigated by classifying those voxels in the 3D image that occupy contacts of chromite grains by whether they fall on chromite-to-chromite contacts (red in Fig. 4) or not (green). We found that, in the UG1 chromitite, 34% of these contact voxels fall on planar contacts, i.e., where chromite grains are in contact with one another along crystal faces (Fig. 4b). If the chromite grains were predominantly in point-to-point contact (Fig. 4a) then this figure would be much lower. The fact that chromite grains are normally touching each other along crystal faces is also evident from direct examination of thin-section photos (Fig. 1d,e) and 3D images (Fig. 2g). Similar textural relations were reported in some earlier studies^43^. Based on this, the chromite chains should have been strong enough to form a self-supporting 3D framework, especially considering that it consists of many billions of interconnected chromite grains in the layer (~ 500,000 grains per 1 cm^3^). In addition, as discussed below, the chromite framework is expected to be locked in place by early growing oikocrysts of plagioclase and orthopyroxene, preventing it from collapse under the weight of the overlying chromite.

Another interesting textural observation is that most chromite grains in the 3D framework of both the planar UG1 and overhanging MR chromitite (which excludes the formation by crystal settling and synneusis) are randomly oriented (Fig. 2; Extended Data Figs. 2 and 4). This is confirmed and quantified using electron backscatter diffraction of our samples (Vukmanovic, unpublished data). This fact has an important implication. It is commonly believed that heterogeneous/self-nucleation should produce aggregates of crystals with preferred crystallographic orientation (e.g., epitaxy) due to surface energy minimization associated with crystallographic alignment of a mineral growing on a substrate^49,50^. Our results suggest that this seems to be not a universal rule for magmatic systems and, at least, in some cases self-nucleation may occur without epitaxial relations between crystals. Thus, both UG1 and MR chromitites appear to be examples of random (non-epitaxial) heterogeneous/self-nucleation of chromite grains on existing crystals at the chamber floor. A mechanism of such random self-nucleation of chromite remains unclear but may likely be related to sudden fluctuations in the degree of kinetic supercooling at a crystal-liquid interface that are caused by a removal of a liquid boundary layer from in situ growing crystals^8,40,51^. We tentatively suggest that the supercooling at a crystal-liquid interface may result in bursts of random nucleation of new crystals against the existing ones because of no time to follow preferred crystallographic orientation.

### A scenario for in situ growth of chromite on the chamber floor

**Figure 5:**
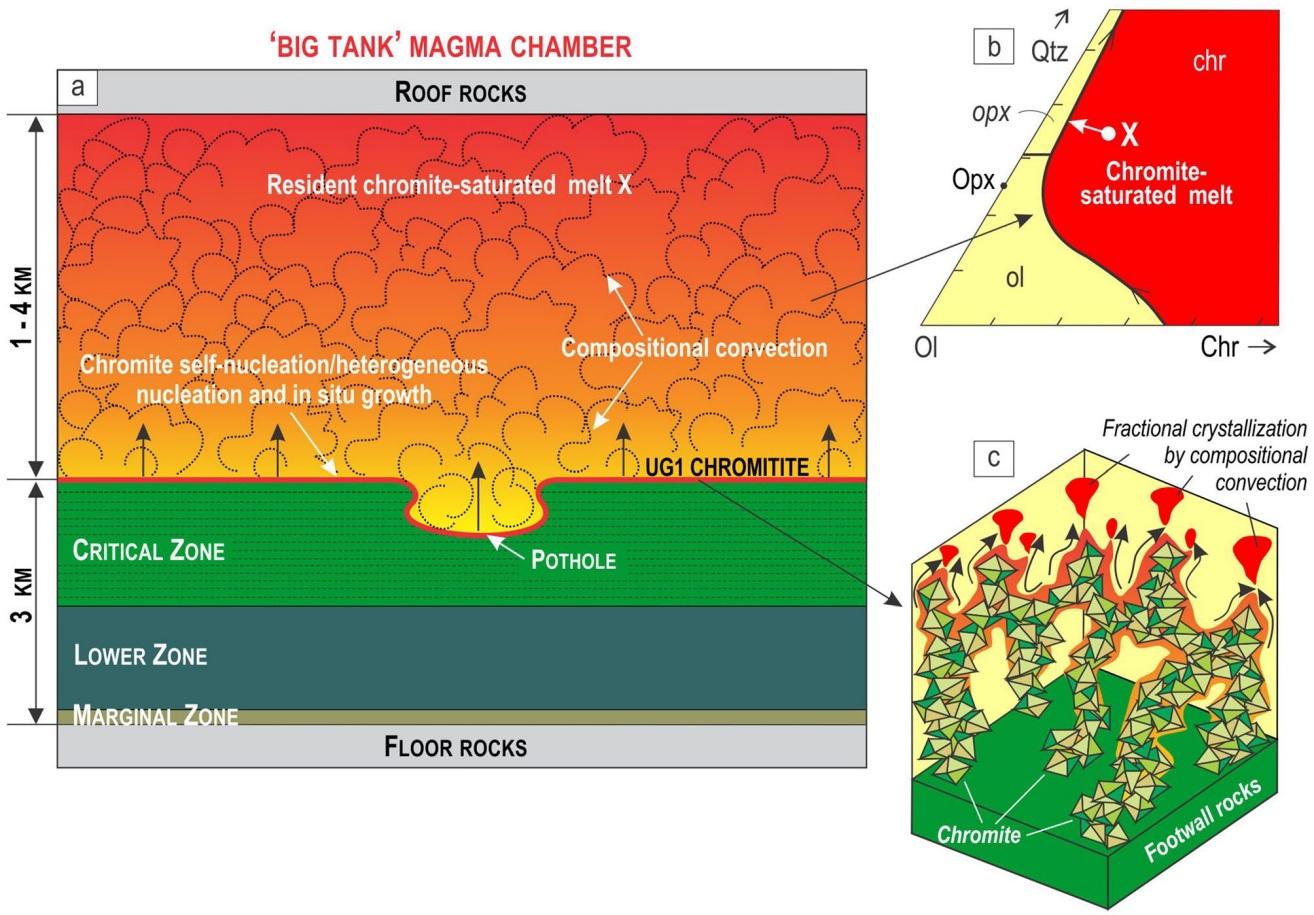
A ‘big-tank’ chamber of the Bushveld Complex filled with a resident melt that crystallizes into the UG1 chromitite at the chamber floor. (**a**) Schematic cartoon of the Bushveld chamber that shows crystallization of the UG1 chromitite near the top of the Critical Zone. The formation of a 1 m thick layer of such chromitite requires an equivalent layer of chromite-only-saturated melt of at least 1 to 4 km in thickness^57,58^. This large volume of liquid may be present in the chamber either as a melt column or as a melt that flows through the chamber for a long period of time. The chromite nucleates and crystallizes directly on the chamber floor, with the resident melt convecting turbulently to deliver Cr for in situ chromite growth. (**b**) Ol–Chr–Qtz phase diagram illustrating the position of a chromite-only-saturated melt parental to the UG1 chromitite. The diagram is modified from reference^39^. Ol, olivine; Opx, orthopyroxene, Chr, chromite; Qtz, quartz. (**c**) A close-up view of the immature UG1 chromitite that forms a 3D framework of touching chromite crystals which self-nucleate on the floor of a magma chamber. Note the low density packing and the low coordination number of chromite in the framework. A compositional boundary layer of buoyant liquid is produced around crystallizing chromite clusters that migrates towards their apex and is released into the overlying melt in the form of compositional plumes, thus causing chemical differentiation in the resident melt. The figure is prepared using CorelDRAW (version 18.1.0.690).

In situ growth of chromite requires crystallisation from a parental melt that was saturated in chromite as the only liquidus phase. Such melts can be produced in response to decompression during their ascent from a deep staging reservoir in which the melts underwent some fractionation and contamination by crustal rocks prior to their ascent^38,52,53^ or by the mixing of a newly injected magma with a resident magma in the chamber along a curved cotectic boundary^32^. We favor here a first scenario because the magma mixing is not consistent with a lack of compositional reversals in the minerals/rocks that overly the chromitite layers^30,37^. In the first scenario, the ascending melts become first slightly superheated relative to its liquidus and then, after some cooling in the chamber, they reach saturation with chromite alone^38^. We proposed that the Bushveld chamber has been replenished by such slightly superheated melts as basal flows that caused thermochemical erosion of the floor cumulates^30^, including the excavation of potholes (Fig. 5a). Our thermodynamic modelling shows that the superheated melts (15 °C above the liquidus) can digest up to 4.5 wt.% of the bulk floor anorthosites without inducing crystallization of the melts, despite them being much colder than the liquidus temperatures of these cumulates. This is equivalent to regional erosion of up to 15 m of the floor cumulates, given a basal melt layer of about 350 m thick^54^. Upon cooling, the melt became saturated in chromite only^38^ (Fig. 5b), with the first chromite grains being nucleated heterogeneously on pre-existing plagioclase crystals of the floor anorthosites. With further cooling, chromite started preferentially self-nucleating, mostly non-epitaxially, on earlier-formed chromite grains to produce composite 3D clusters which subsequently merged into a continuous 3D framework of touching, randomly oriented chromite grains (Fig. 5c). New crystals emerged in the system mostly by heterogeneous/self-nucleation because the activation energy for this process is much lower relative to other types of nucleation^8,39^.

**Figure 6:**
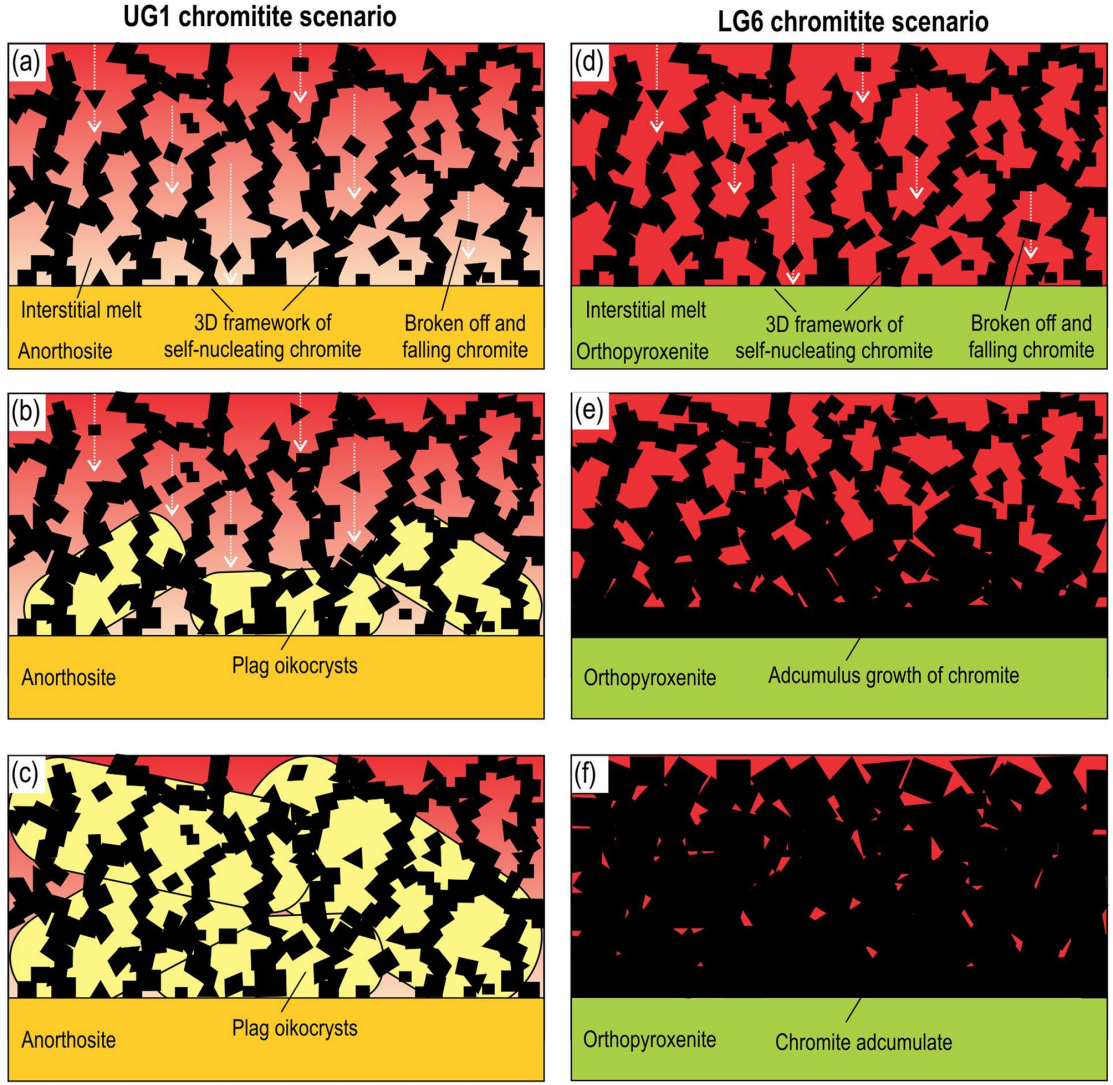
Two contrasting scenarios for the textural evolution of a 3D framework of chromite crystals in massive chromitites of the Bushveld Complex. (**a–c**) UG1 chromitite starts forming as a 3D framework of touching chromite crystals (> 500,000 grains per 1 cm^3^) which self-nucleate on the anorthosite floor of a magma chamber. This initial framework has been preserved owing to the early growth of plagioclase (and pyroxene) oikocrysts from an interstitial melt. The early nucleation of plagioclase/pyroxene oikocrysts is likely due to a short interval of chromite-only crystallization of the parental melt. (**d–f**) LG6 chromitite also starts forming as a 3D framework of touching chromite crystals which self-nucleate on the orthopyroxenite floor of a magma chamber. However, in this case the initial framework has been obliterated by adcumulus growth of chromite (up to 100% chromite) which was not arrested by crystallization of plagioclase/pyroxene oikocrysts. No nucleation and growth of silicate phases oikocrysts is likely due to a much longer interval of chromite-only crystallization of the parental melt. In both scenarios, the growth of a 3D framework of touching chromite crystals has been accompanied by settling of chromite grains that grew in situ at the crystal-liquid interface but were torn loose by flowing melt and collapsed downwards into the open space of the crystal framework (a & d). Some of the grains have landed on and were captured by growing oikocrysts (b) and, therefore, occur there as entirely discrete grains. Crystal sizes are not to scale. The figure is prepared using CorelDRAW (version 18.1.0.690).

We envisage that chemical differentiation of the resident melt in the chamber at that time occurred by convective removal of a buoyant compositional boundary layer^55^ from in situ growing chromite crystals in a 3D framework (Fig. 5c). The boundary layer becomes buoyant relative to its parental melt because chromite (spinel) fractionation results in a strong decrease in the density of the melts^41,56^. The differentiation is aided by high porosity and permeability of a 3D crystal framework that permits the easy chemical exchange of melts between the crystal framework and the main magma body. New chromite grains successively emerge at the crystal-liquid interface due to strong kinetic supercooling that occasionally occurs there in response to a convective removal of a compositional boundary layer from in situ growing chromite crystals^40^. The fluctuations in the degree of kinetic supercooling are likely responsible for the non-epitaxial relations between self-nucleating chromite grains. The remarkable preservation of the nucleation/growth history in the UG1 chromitite is due to the early growth of plagioclase and pyroxene oikocrysts that have ‘frozen in’ a 3D chromite framework at its early immature stage (Fig. 6a–c). The early nucleation and growth of plagioclase/pyroxene oikocrysts is likely due to a relatively short interval of chromite-only crystallization of the parental melt. The plagioclase oikocrysts are thought to grow from the base of a chromitite layer because of the possibility of their self-nucleation on the plagioclase primocrysts of anorthosites. A small portion of crystals (3 vol.%) that occurs as entirely discrete grains (Fig. 2d) are likely those which initially grew at a crystal-liquid interface but were torn loose by flowing melt, collapsed downwards into the open space of the crystal framework (Fig. 6a) and were captured by upward-growing oikocrysts (Fig. 6b and c).

This continuous 3D framework of chromite grains (Fig. 2) is thus a remarkable example of a natural solidification front which develops via self-nucleation/heterogeneous nucleation^40^. The 3D plagioclase frameworks described by Philpotts et al. in dolerites, although similar in form, are thought to have formed by other mechanisms^47^. Under other circumstances, the initial chromite framework would have evolved into perfect chromitite adcumulate (up to 100% chromite; e.g., LG chromitite in Extended Data Fig. 5) in which all primary information is lost (Fig. 6d–f). In this case, no nucleation and growth of plagioclase/pyroxene oikocrysts that could have prevented the adcumulus growth is likely due to a much longer interval of chromite-only crystallization of the parental melt. We propose that most layers of monomineralic chromitites in the Bushveld Complex likely started their life as porous, in situ produced 3D chromite frameworks that can no longer be seen due to adcumulus overgrowth^59^. We further propose that the observed shape of the coordination number vs grain size curve (Fig. 3c) may be diagnostic of chromite cumulates formed by in situ crystallisation involving heterogeneous self-nucleation^30,31^.

### Alternative interpretations to the UG1 chromitite and its texture

#### Models involving reactive fluid infiltration from underlying cumulates

Chromitites in layered intrusions, including the Bushveld Complex, have been interpreted as a product of metasomatic processes involving a reaction of upward-migrating volatile-rich fluids or interstitial melts with pre-existing noritic cumulates^60–63^. In this model, the 3D chromite framework (Figs. 1–3) would have to be attributed to post-nucleation formation taking place during crystal aging^64^. However, this concept has so far only been applied to explain the origin of very thin (mm to cm) chromitite seams that are locally developed along the contacts of some cumulate layers^60,62,65^. A key challenge to this concept is thus to explain the enormous lateral extent and mineralogical uniformity of thick chromitite layers^28^ (e.g., Fig. 1b). In particular, the metasomatic models need to elucidate how mostly vertically channelized fluids/melts^66^ may produce a 1–2 m thick chromitite layer (e.g. UG1 or UG2) that extends laterally over distances of up to 400 km (Fig. 1a) and how this process may result in the identical vertical distribution of platinum-group elements across the chromitites that are separated laterally by over 300 km^28^. In addition, the metasomatic models need to be reconciled with abundant field and textural observations on massive chromitites indicating their physical deposition on the chamber floor from the overlying magma^30,41,67^. Even more importantly, the theoretical predictions of metasomatic models^60^ that noritic cumulates can be metasomatically transformed into thick and extensive layers of massive chromitites need to be supported by field and textural observations from layered intrusions. Without such direct evidence, the metasomatic models will remain incomplete and can hardly be considered as a viable mechanism for the origin of stratiform chromitites in layered intrusions.

#### Models involving remelting of cumulates by hot/volatile-rich melts

In this group of models, gabbroic, noritic or orthopyroxenitic cumulates are subjected to remelting by hot magma (regarded by some authors as sills intruding into pre-existing cumulates) that produces a chromite-only-saturated melt subsequently crystallizing chromitite seams. Again, only very thin (mm to cm thick) chromitite seams have so far been attributed to this process^22,68,69^ and it remains, therefore, to be shown if this idea can be extended to explain the origin of the 1–2 m thick and 400 km long layers of stratiform chromitites (Fig. 1a,b). One recent attempt to do this, although not involving magma emplacement, has been undertaken by Veksler and Hou^70^. Their model envisages the generation of a chromite-only saturated melt by large-scale hydration melting of the Cr-rich orthopyroxenite cumulates at the chamber floor. Melting is proposed to have been triggered by addition of 3–4 wt.% H_2_O to a stagnant layer of a dry melt at the base of the magma chamber by hydrous fluids derived from underlying sediments of the Bushveld Complex. Hydration melting of cumulates is accompanied by in situ crystallization of chromite on the uneven erosional surface from a basal stagnant layer. The idea is attractive because it may explain the UG1 chromitite texture (Figs. 1–3) in the same way as proposed here. However, the model appears to be physically unrealistic. The study itself shows that such amount of water will cause a dramatic decrease in dry melt density (from 2607 kg/m^3^ to 2429 kg/m^3^ at 1125 °C and 300 MPa)^70^ making a basal layer highly buoyant. As a result, the basal layer will be flushed away by compositional convection, thereby precluding hydration melting of floor cumulates and formation of a chromite-only-saturated melt. In fact, there seems to be even more likely that fluids arriving into the chamber through fractures in solidified cumulates will have no time to dissolve in the basal layer at all, but would rather be immediately transported into and mixed with the entire main magma body^66^. In addition, this idea cannot be applied to our case of the UG1 chromitite because this layer is closely associated with anorthosites (Fig. 1b) that are very poor in Cr and their potential melting will be of little help in generation of a chromite-only-saturated melt.

#### Models involving emplacement of crystal-rich mushes

In this group of hypotheses, the massive chromitites are produced from crystal-rich mushes by physical separation of chromite from coexisting silicate minerals^36,37,53,71,72^. One of the latest ideas along this line is generation of the Bushveld chromitites from late-stage sills of crystal-rich slurries that have intruded the pre-existing noritic rocks of the Critical Zone and formed mafic–ultramafic units^16,19^. According to this model chromite was segregated from coexisting pyroxene crystals by kinetic sieving and accumulated at the base of the slurry while it was still flowing along the sill floor. Our data show, however, that this model (and other gravitational concepts^36,37,53,71^) is not consistent with textural observations (Figs. 1–3) indicating no gravity settling of individual chromite grains. Mungall (pers. comm., 2021) and Bédard (pers. comm., 2021) have suggested, however, an interesting way to overcome this obstacle. They pointed out that the spatial arrangement of chromite in the UG1 (Figs. 1–3) is similar to what is observed in rocks where chromite co-precipitates with olivine or pyroxene^73^. On this basis, they suggested that the UG1 chromitite layers (Fig. 1b) were originally olivine-chromite or orthopyroxene-chromite cumulates deposited from crystal-rich mushes on the chamber floor. Subsequently, these cumulates were, however, completely replaced by the plagioclase oikocrysts as if cumulus olivine/orthopyroxene were not even there. For this reason, the chromite grains are now apparently suspended in an assemblage of plagioclase oikocrysts (Figs. 1, 2, 3). We acknowledge the high originality of this idea but cannot subscribe to it because of the current lack of any supporting field and textural evidence for the UG1 chromitite being originally the olivine-chromite or orthopyroxene-chromite cumulates. If this were really the case, the remnants of the original olivine/orthopyroxene protoliths would almost certainly be reported in some of the numerous papers/theses dealing with the UG1 chromitite. The model also faces some other serious problems. In particular, reliable field and textural evidence has not yet been presented for the mafic–ultramafic units (e.g., UG1 and UG2) to be true late-stage sills^16,19,20^. The only evidence that has been reported so far is the out-of-sequence ages of zircons from these units^16,20^. However, these absolute ages are now shown to be at odds with the relative ages of rocks as defined by cross-cutting relations in potholes of the Bushveld Complex^74^. In contrast, there are field and textural observations that appear to be at variance with this concept. Among them is the lack of crosscutting relationships of the units with overlying host rocks, no internal chilled margins of the units against these rocks, identical chemical composition of minerals in the units and the adjacent rocks^74–76^. Finally, this concept needs to be reconciled with numerous field and textural observations on massive chromitites^30,41,67,77^, in particular, with their development on overhanging sidewalls of potholes indicating the formation of chromitites by in situ crystallization^30^.

### Chromium budget requires a large magma volume

The realization that massive chromitites form by in situ growth of chromite directly in the chamber (Fig. 5a)—rather than from chromite phenocrysts brought into the chamber with externally-derived crystal-rich mushes^16,19,35,37,78^—logically brings us to a long-known Cr mass balance issue^58,71,79^. The stratiform chromitite layers in layered intrusions can be up to 2 m thick and contain 40–50 wt.% Cr_2_O_3_, yet have evidently crystallized from a basaltic melt that was unlikely to have contained more than 1000 ppm Cr^57^. An implication is that the formation of a thick chromitite layer, such as the UG1 chromitite, requires extraction of Cr from a very large volume of liquid that can be present either as a thick melt layer in the chamber or as the melt flowing through the chamber, or both. To illustrate, given the thickness of a chromite layer, from mass-balance considerations one can calculate the volume of the parental melt in terms of the thickness of an equivalent layer. If a chromite layer crystallized at 1250 °C and the *f*O_2_ equivalent to QFM buffer, then based on experimental data^57,79^, the chromite should contain ~ 45% Cr_2_O_3_, and the coexisting melt should carry about 0.10 wt% Cr_2_O_3_. Assuming that chromitites formed from the overlying melt, it can be estimated that a 1 m thick layer of chromitite will require a magma column of about 4 km thick (Fig. 5a). The thickness can be reduced to 2 km^58^ or 1 km^28^ if Cr solubility in a parental melt is to increase by its higher temperature or lower *f*O_2_^58^. These estimations assume 30% of the Cr removal from a parental melt^58^. One cannot remove any more Cr from the melt than that because otherwise the melt will reach a cotectic with other liquidus phases (e.g., olivine or orthopyroxene) terminating chromitite formation (Fig. 5b).

This one-dimensional modelling illustrates the mass-balance issue. Applying this logic to the Bushveld Complex, it has been estimated that the formation of its most prominent chromitites would have required a column of 13 to 15 km of a parental chromite-saturated liquid^26,28^. In addressing this mass-balance requirement, Cawthorn and Walraven^26^ modelled the Bushveld chamber as a long-lived flow-through system (~ 75,000 years life-time) that developed via a large number of injection events, partial crystallization of these magma batches, and removal of their residual liquids from the chamber by the succeeding magma batches. They concluded that the total volume of basaltic magma involved was 740–1200*10^3^ km^3^, with only ~ 50% of this being represented by the cumulates now seen within the Bushveld Complex^26^.

There are two common misconceptions regarding the destiny of the escaped residual melt that needs to be mentioned here. First, it is believed that this melt must be present as basalt lavas above the Bushveld Complex^71,80^ and, second, these basalts must be strongly depleted in Cr. Regarding the first issue, recent seismic reflection and field-based studies indicate that most magma moves through the lithosphere mostly sideways as interconnected sill complexes rather than upwards as vertical dykes^81^. It has been shown that such sill complexes can facilitate the magma transport over lateral distances as much as ~ 4100 km^82^. If so, the Bushveld-related volcanoes fed by such sill complexes should not necessarily overlie the Bushveld Complex (i.e., melt source). Rather, the magma that went through the Bushveld chamber may have ended up many hundreds if not thousands of km away as lateral sills or lavas which can be subsequently entirely eroded away. Therefore, the chances to find the lavas/sills formed from the Bushveld-related magmas are exceedingly low. One place where the magma that laterally^28^ escaped from the Bushveld chamber (not necessarily at the time of the chromitite formation) has been fortunate to get preserved is the Molopo Farms Complex located about 200 km west of the Bushveld Complex^83,84^. Regarding the second issue, the rocks that may be produced from the escaped magma (in sills/intrusions) should not necessarily be depleted in Cr. This is because even after formation of chromitite layers the melts saturated in chromite only, these melts may still be saturated in chromite due to their location on some chromite-silicate mineral cotectics (e.g., Fig. 5b). It is therefore not surprizing, for example, that cumulates of the Molopo Farms Complex show no depletion in Cr^83,84^.

### The classical magma chamber paradigm is still alive!

The enormous lateral extent of in situ formed chromitite layers and related mass-balance considerations indicate that during the formation of massive chromitites the Bushveld chamber was operating as a giant magma body of more than 400 km in diameter, with a column of the resident melt likely attaining a few km in thickness. Thus, starting from this stage the Bushveld Complex has most likely been developed as a large, long-lived and predominantly molten magma chamber (a true ‘big tank’ reservoir) in Earth’s crust (Fig. 5a). The conclusion is further supported by the remarkable homogeneity of Sr isotopes over an interval of more than 2.5 km of the Upper Zone^85^, which indicates a melt column thickness in the chamber being that thick or even thicker^86,87^. This is in contrast with some recent models, mostly based on out-of-sequence geochronology^16,20^, that depict this giant complex as a stack of thin crystal-rich sills^16,18,20^. Field relationships revealed, however, some serious problems with interpretation of zircon isotopic ages in these studies^74^. Our inference may be likely extended to some other large mafic–ultramafic layered intrusions that contained thick and laterally extensive layers of monomineralic chromitites (e.g., Stillwater and Great Dyke). It should be stressed that such intrusions are quite rare through the whole of geological time, so it is not surprising that there are no known examples of equivalent magma chambers that are active and detectable in the present-day Earth’s crust^12,15^. We conclude that it is too early to discard the classical paradigm of a magma chamber developed by several generations of petrological luminaries^1–5,24^. Rather, we suggest re-directing our efforts to find out how new geophysical, geochronological and thermal/diffusion modelling^10–20^ can be logically reconciled with the classical paradigm.

## Methods

### Rock sampling and petrography

Documentation of field observations of the UG1 chromitite was undertaken at the Dwars River locality and its sampling at the nearby Mototolo Mine from the HEX 076 drill-core in the Eastern Bushveld Complex. Thin sections and polished blocks were cut from orientated sample blocks to be as close as possible to the original vertical position. Thin sections and polished blocks were studied using a polarised light microscope with a circular stage and photographed using the Olympus 224 BX-63 OM/FM optical microscope housed at the MMU (Microscopy and Microanalysis Unit) of the University of the Witwatersrand, Johannesburg, South Africa.

### High resolution X-ray computed tomography and 3D image analysis and quantification

The UG1 sample was scanned using the Zeiss Versa XRM 520 3D x-ray microscope installed at the Australian Resources Research Centre (CSIRO Mineral Resources, Kensington, Western-Australia). The instrument was set to maximize the contrast between chromite and silicates (plagioclase and pyroxene) present in the sample. Two scans at a voxel size of 5 µm were performed along the vertical axis of the samples and were stitched in 3D to maximize the volume of sample used for further analysis. A total of 1601 projections were recorded over 360° degrees rotation for each scan and were used for volume reconstruction. Beam hardening and ring artefacts were minimized during data acquisition and corrected (if necessary) during image reconstruction. After reconstruction, the sample is represented by a regular grid (1998 × 2046 × 3748 voxels) where each voxel has a unique greyscale value. Chromite, plagioclase and pyroxene were segmented from the volumes using a 3D gradient watershed algorithm^88^ to produce binary images. The separation of touching chromite crystals in 3D was done using a modified version of the algorithm used to separate touching chromite in komatiites^89^ and chromite from the normal Merensky Reef^90^ using Avizo2020 and Matlab software. The shape and size characteristics of chromite network and individual grains were computed to provide quantitative measure of chromite grains above 15 µm equivalent sphere diameter (ESD). Chromite grains were defined as touching each other using an 18-voxel connectivity threshold (i.e., voxels are connected if their faces or edges touch). The coordination number of each chromite grains (i.e., the total number of other chromite grains touching in 3D a given grain) was also calculated. All results are summarized in Figs. 2 and 3.

### Random packing simulation

The simulated Random Packing of UG1 chromite was generated using the Discrete Element Method as described in ref^91^. The individual chromite particles were modelled as spheres with a size distribution as measured from the UG1 chromite sample ranging from 15 µm to 420 µm, and an interparticle friction coefficient of 0.9. The simulation box has dimensions 5 mm by 2 mm by 2 mm, with periodic boundaries in the two directions normal to gravity. Particles are initially distributed randomly in the simulation box and then allowed to slowly settle under gravity subject to a Stokes’ drag force to form a random loose packing^44^. The packing is then analysed to determine the packing density and the distribution of inter-particle contacts between particles.

## Supplementary Information

Supplementary Information 1.

Supplementary Information 2.

Supplementary Video 1.

Supplementary Video 2.

## Data Availability

The authors declare that all relevant data are available within the article and its Supplementary Information Files.
