# Supplementary material for: Chromitite layers indicate the existence of large, long-lived, and entirely molten magma chambers
Source: Sci Rep. 2022 Mar 8;12:4092. doi: 10.1038/s41598-022-08110-6 (PMC8904791; doi:10.1038/s41598-022-08110-6)
Supplement: Supplementary file 1 — Supplementary Information 1. [file 41598_2022_8110_MOESM1_ESM.pdf]

Extended data for a paper entitled

“Chromitite layers indicate the existence of large, long-lived, and entirely molten magma chambers”

by Latypov et al. (2022)

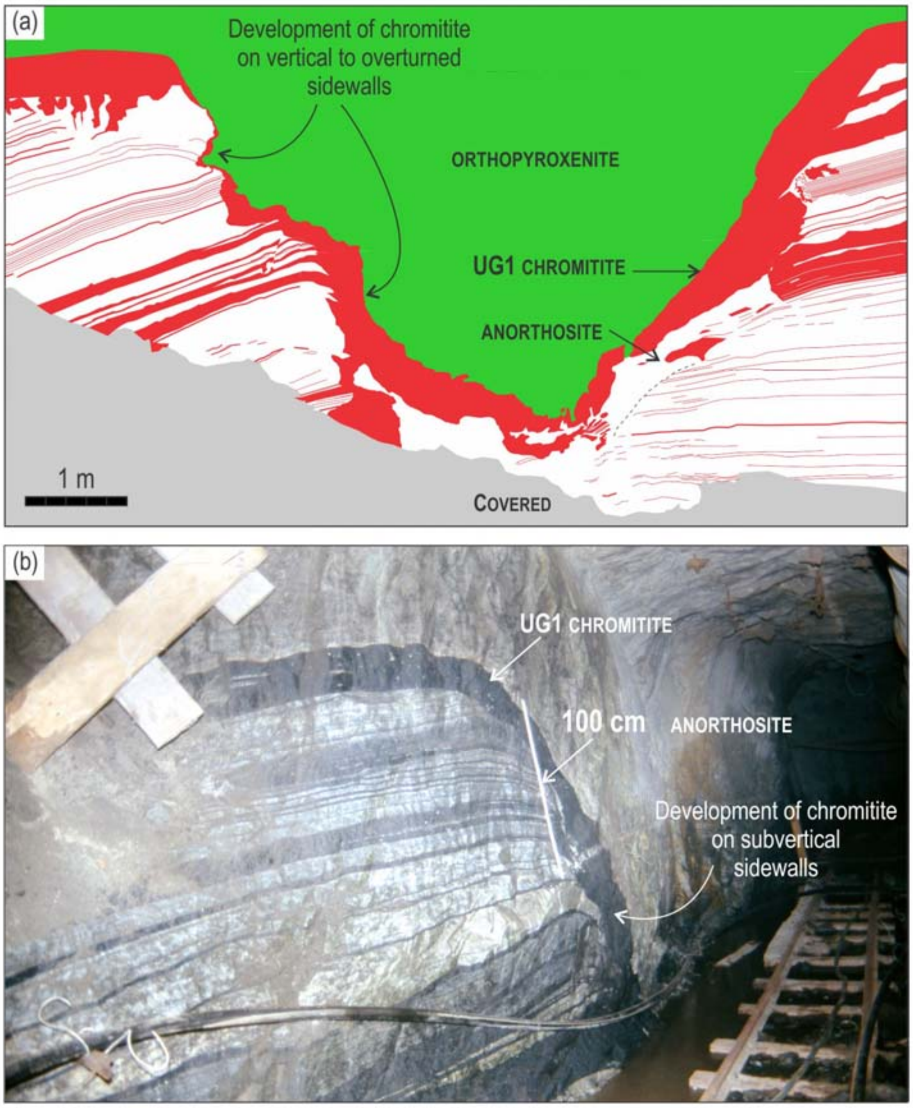

Extended Data Fig. 1. Field observations that indicate *in situ* crystallization of the Upper Group 1 (UG1) chromitite in the Bushveld Complex, South Africa. (a) Sketch of an underground exposure showing the UG1 chromitite that drapes over steeply inclined to overhanging walls of a pothole. Noteworthy feature is the crosscutting relationships of the UG1 chromitite with interlayered anorthosite and chromitite in the footwall rocks. Level 15, Shaft 10 of the Impala Platinum Mine, Western Bushveld. (b) Photograph of a pothole with the UG1 chromitite that drapes over the floor with well-developed interlayering of anorthosite and chromitites in a domal structure. It should be noted that the UG1 chromitite develops along the subvertical walls without any notable change in its thickness. Level 13, Rustenburg Platinum (Brakspruit Mine), Western Bushveld. Such a position of the UG1 chromitite indicates its formation by *in situ* crystallization directly at the chamber floor. The figure (a) is prepared using CorelDRAW (version 18.1.0.690).

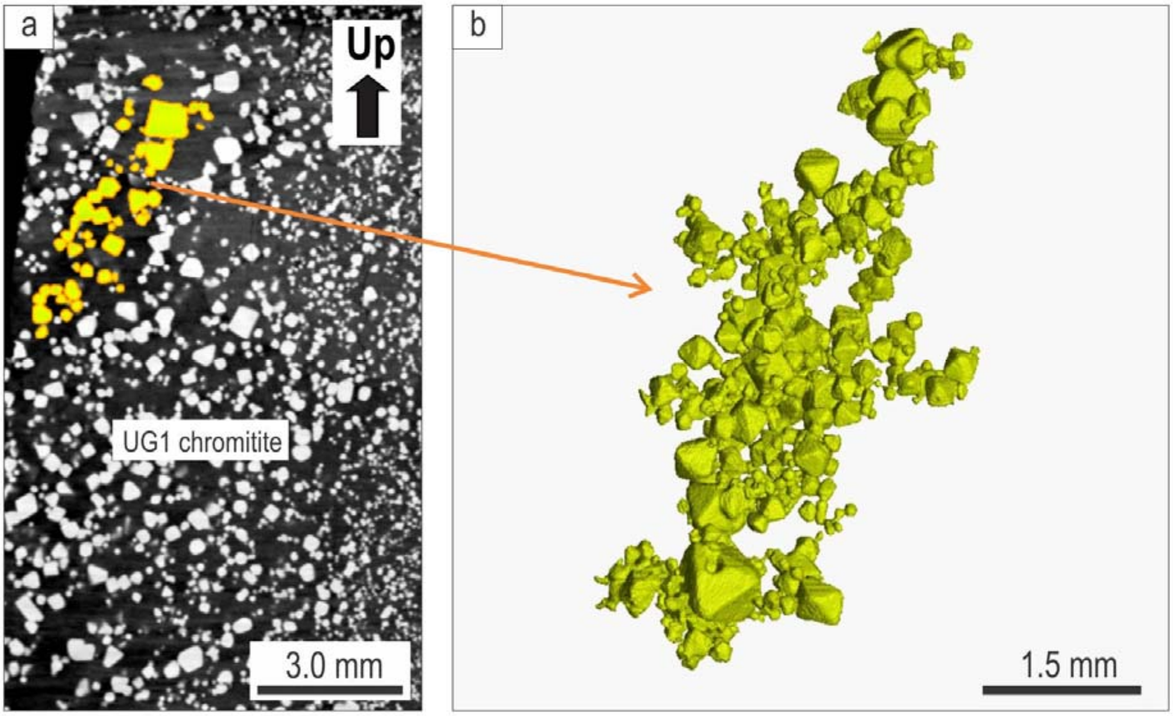

Extended Data Fig. 2. High-resolution X-ray computed tomography images revealing that nearly all chromite grains in UG1 chromitite are linked into a continuous 3D framework. **a,** Image indicating a position of a chromite framework (yellow grains) shown in (b) from the planar UG1 chromitite. Cumulus chromite is white while silicate minerals, mostly oikocrysts of plagioclase, are grey to black. Sample HX-07-153.33, Mototolo mine, Eastern Limb. **b,** Image showing that touching chromite crystals are bound together into a continuous 3D framework and are, therefore, not free to settle towards the base of the UG1 chromitite layer. This relationship between chromite crystals is most clearly seen from a 3D animation (Supplementary Video 1). The figure is prepared using ImageJ (version 1.8.0).

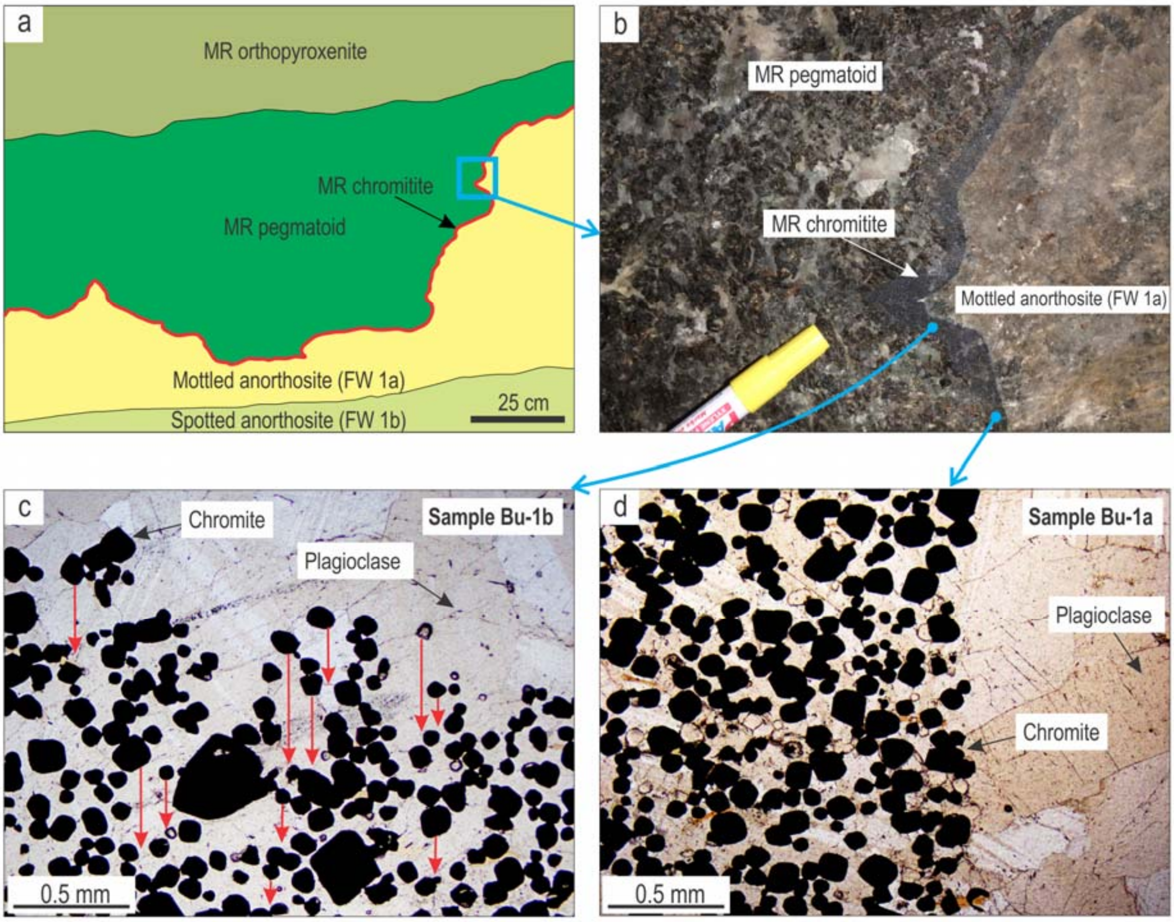

Extended Data Fig. 3. A notable lack of field and textural evidence for gravity settling of chromite crystals within an overhanging chromitite seam of the Merensky Reef (MR). (a) Sketch of a pothole with the MR extending from the steep sidewall into footwall mottled anorthosite. Note that a chromitite seam developed along the basal contact of MR pegmatoid. (b) Close-up photograph showing the MR with a chromitite seam that drapes over vertical to overhanging sidewalls of the pothole. Such a position of the seam indicates its formation by *in situ* crystallization. (c) Photograph of a thin-section (under plane polarized light) of the MR chromitite showing isolated chromite grains and their loose clusters enclosed by oikocrysts of plagioclase, a sample Bu-1b. Red arrows emphasize that chromite grains show no tendency to gravitate despite the mush is highly porous (~60 vol.%); (d) Photograph of a thin-section (under plane polarized light) of the MR chromitite showing isolated chromite grains and their loose clusters mostly enclosed by oikocrysts of plagioclase, a sample Bu-1a. Shaft 3 of the Karee mine (previously owned by Lonmin Platinum and now owned by Sibanye-Stillwater), Western Bushveld Complex. The figure (a) is prepared using CorelDRAW (version 18.1.0.690).

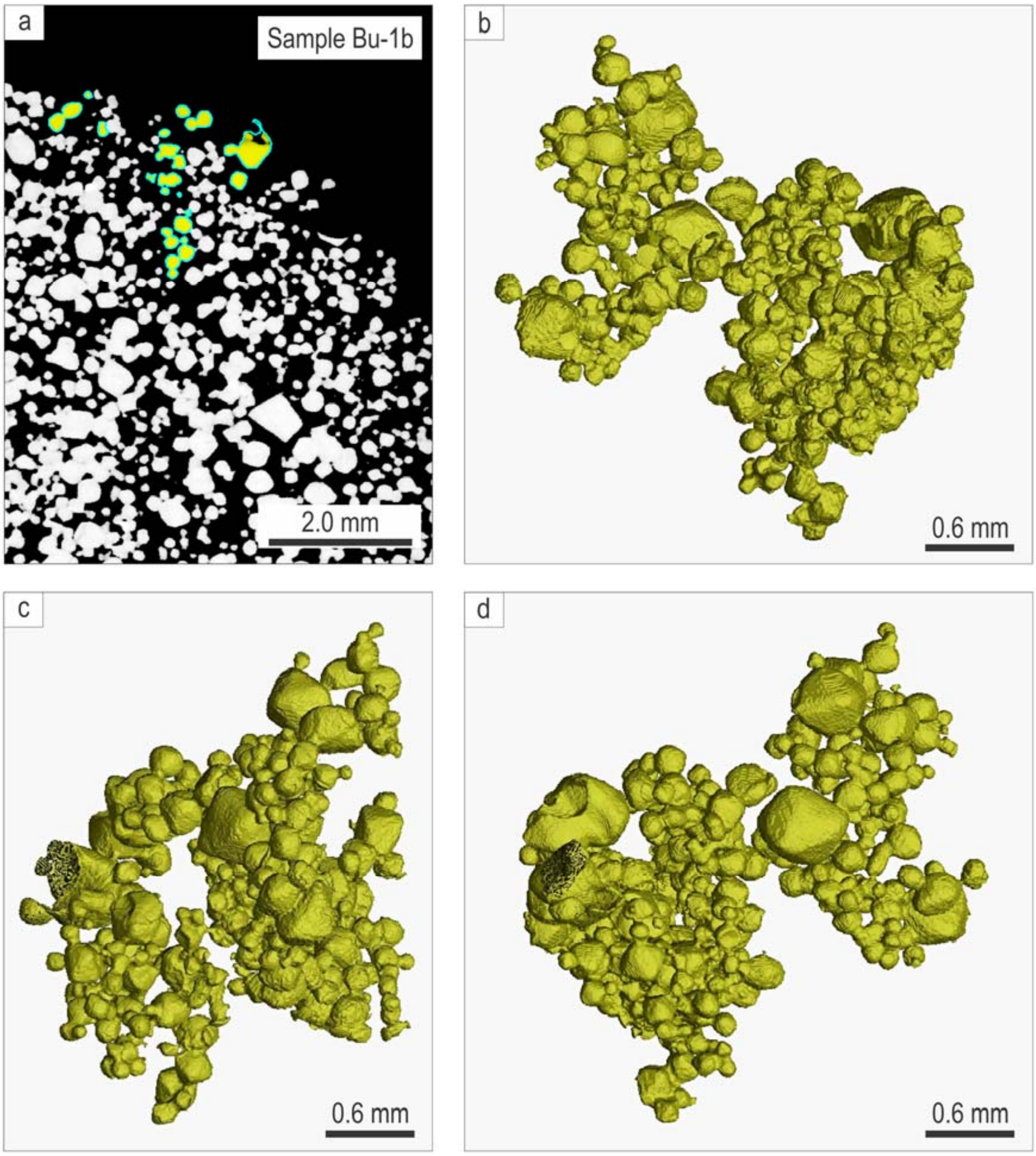

Extended Data Fig. 4. High-resolution X-ray computed tomography images revealing that all chromite grains in a chromitite seam of the Merensky Reef are linked into a continuous three-dimensional framework. (a) Image indicating a position (yellow grains) of a chromite framework shown in (c-d) from the studied MR chromitite, a sample Bu-1b. Cumulus chromite is white while silicate minerals, mostly oikocrysts of plagioclase, are black. (b-d) Images showing that touching chromite crystals (yellow) are bound together into a continuous 3D framework and are, therefore, not free to settle downwards. This relationship between chromite crystals is most clearly seen from a 3D animation (Supplementary Video 2). The figure is prepared using ImageJ (version 1.8.0).

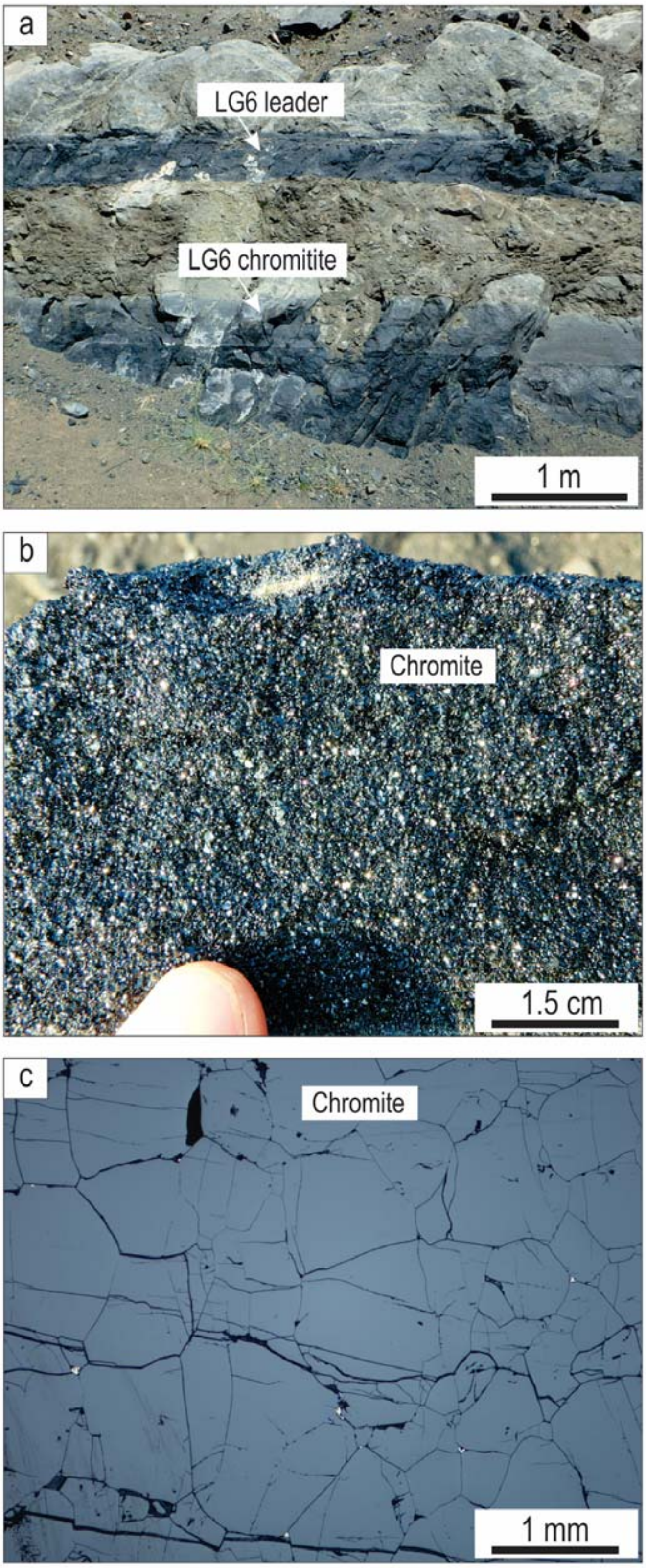

Extended Data Fig. 5. LG6 chromitite of the Bushveld Complex in which the original 3D framework of touching chromite crystals has been likely erazed by adcumulus overgrowth.

(a) Field photograph of massive LG chromitite and its leader from the Critical Zone. Jagdlust area, Eastern Bushveld Complex. (b) A hand specimen of LG6 chromitite indicating its almost monomineralic composition. Mecklenburg Chrome Mine, Eastern Bushveld Complex. (c) Photomicrograph in reflected light of the LG6 chromitite from the 13R-9 drill core, Ruighoek Chrome Mine, Western Bushveld Complex. Notice the nearly-perfect adcumulate nature of the chromitite with virtually no intercumulus phases. No primary information on crystal nucleation and growth in preserved in this sample due its adcumulate nature.
